# Supplementary material for: Multi‐dataset identification of innovative feature genes and molecular mechanisms in keratoconus
Source: J Cell Mol Med. 2024 Sep 19;28(18):e70079. doi: 10.1111/jcmm.70079 (PMC11412914; doi:10.1111/jcmm.70079)
Supplement: Supplementary file 2 — Material S2. [file JCMM-28-e70079-s002.zip › 7.GSEA analysis/4.MYRF/KEGG_ADHERENS_JUNCTION.html]

Details for gene set KEGG\_ADHERENS\_JUNCTION[GSEA]

|  || Dataset | MYRF.group.cls#h\_versus\_l |
| Phenotype | group.cls#h\_versus\_l |
| Upregulated in class | l |
| GeneSet | KEGG\_ADHERENS\_JUNCTION |
| Enrichment Score (ES) | -0.41018558 |
| Normalized Enrichment Score (NES) | -1.5709319 |
| Nominal p-value | 0.077844314 |
| FDR q-value | 0.21696882 |
| FWER p-Value | 0.657 |
Table: GSEA Results Summary

  

Fig 1: Enrichment plot: KEGG\_ADHERENS\_JUNCTION      
 Profile of the Running ES Score & Positions of GeneSet Members on the Rank Ordered List

  

| SYMBOL | TITLE | RANK IN GENE LIST | RANK METRIC SCORE | RUNNING ES | CORE ENRICHMENT || 1 | RAC3 | na | 2468 | 0.325 | -0.0290 | No |
| 2 | IGF1R | na | 7135 | 0.160 | -0.1428 | No |
| 3 | FARP2 | na | 7986 | 0.139 | -0.1494 | No |
| 4 | BAIAP2 | na | 9266 | 0.110 | -0.1720 | No |
| 5 | PTPRJ | na | 9317 | 0.109 | -0.1593 | No |
| 6 | CREBBP | na | 9688 | 0.102 | -0.1567 | No |
| 7 | CSNK2A1 | na | 10382 | 0.089 | -0.1652 | No |
| 8 | CTNND1 | na | 11572 | 0.069 | -0.1905 | No |
| 9 | SORBS1 | na | 12393 | 0.058 | -0.2066 | No |
| 10 | ACTB | na | 12485 | 0.057 | -0.2018 | No |
| 11 | IQGAP1 | na | 12574 | 0.056 | -0.1971 | No |
| 12 | TJP1 | na | 12904 | 0.051 | -0.2000 | No |
| 13 | ERBB2 | na | 13228 | 0.047 | -0.2032 | No |
| 14 | SMAD4 | na | 13356 | 0.045 | -0.2009 | No |
| 15 | ACP1 | na | 13480 | 0.044 | -0.1988 | No |
| 16 | INSR | na | 14026 | 0.038 | -0.2096 | No |
| 17 | LMO7 | na | 14276 | 0.035 | -0.2123 | No |
| 18 | MAPK3 | na | 14382 | 0.034 | -0.2109 | No |
| 19 | CTNNA2 | na | 14571 | 0.032 | -0.2122 | No |
| 20 | WAS | na | 14632 | 0.031 | -0.2099 | No |
| 21 | PTPRF | na | 14783 | 0.030 | -0.2104 | No |
| 22 | ACTN4 | na | 14881 | 0.029 | -0.2094 | No |
| 23 | TCF7L2 | na | 15138 | 0.027 | -0.2134 | No |
| 24 | CTNNB1 | na | 15600 | 0.022 | -0.2238 | No |
| 25 | CDH1 | na | 15708 | 0.021 | -0.2242 | No |
| 26 | NLK | na | 16181 | 0.016 | -0.2358 | No |
| 27 | PARD3 | na | 16431 | 0.013 | -0.2412 | No |
| 28 | CTNNA1 | na | 16494 | 0.013 | -0.2413 | No |
| 29 | EGFR | na | 17031 | 0.008 | -0.2557 | No |
| 30 | RAC2 | na | 17259 | 0.006 | -0.2614 | No |
| 31 | WASL | na | 17383 | 0.005 | -0.2643 | No |
| 32 | SMAD3 | na | 17747 | 0.002 | -0.2745 | No |
| 33 | SMAD2 | na | 18091 | -0.001 | -0.2843 | No |
| 34 | WASF2 | na | 18382 | -0.004 | -0.2921 | No |
| 35 | RHOA | na | 18827 | -0.008 | -0.3038 | No |
| 36 | CSNK2B | na | 19992 | -0.022 | -0.3346 | No |
| 37 | MAPK1 | na | 20406 | -0.027 | -0.3430 | No |
| 38 | CSNK2A2 | na | 20546 | -0.028 | -0.3434 | No |
| 39 | RAC1 | na | 21936 | -0.045 | -0.3777 | No |
| 40 | ACTG1 | na | 22317 | -0.050 | -0.3822 | No |
| 41 | PTPN1 | na | 22461 | -0.052 | -0.3796 | No |
| 42 | WASF1 | na | 23182 | -0.062 | -0.3923 | No |
| 43 | YES1 | na | 23276 | -0.064 | -0.3867 | No |
| 44 | CDC42 | na | 23297 | -0.064 | -0.3789 | No |
| 45 | VCL | na | 24000 | -0.076 | -0.3893 | No |
| 46 | MAP3K7 | na | 24724 | -0.088 | -0.3987 | Yes |
| 47 | TGFBR2 | na | 24993 | -0.093 | -0.3944 | Yes |
| 48 | CTNNA3 | na | 25302 | -0.099 | -0.3905 | Yes |
| 49 | PTPN6 | na | 25517 | -0.103 | -0.3833 | Yes |
| 50 | TGFBR1 | na | 25528 | -0.103 | -0.3702 | Yes |
| 51 | FER | na | 25539 | -0.103 | -0.3570 | Yes |
| 52 | SNAI1 | na | 26441 | -0.122 | -0.3672 | Yes |
| 53 | WASF3 | na | 27298 | -0.142 | -0.3734 | Yes |
| 54 | EP300 | na | 27396 | -0.145 | -0.3574 | Yes |
| 55 | TCF7L1 | na | 27666 | -0.152 | -0.3454 | Yes |
| 56 | ACTN3 | na | 27890 | -0.157 | -0.3315 | Yes |
| 57 | ACTN1 | na | 27985 | -0.160 | -0.3134 | Yes |
| 58 | MET | na | 28755 | -0.183 | -0.3118 | Yes |
| 59 | SSX2IP | na | 29508 | -0.206 | -0.3067 | Yes |
| 60 | PTPRM | na | 29667 | -0.212 | -0.2838 | Yes |
| 61 | TCF7 | na | 30107 | -0.227 | -0.2669 | Yes |
| 62 | ACTN2 | na | 30149 | -0.229 | -0.2384 | Yes |
| 63 | SRC | na | 31183 | -0.273 | -0.2327 | Yes |
| 64 | SNAI2 | na | 32352 | -0.339 | -0.2224 | Yes |
| 65 | PTPRB | na | 32753 | -0.369 | -0.1861 | Yes |
| 66 | LEF1 | na | 33408 | -0.432 | -0.1489 | Yes |
| 67 | FYN | na | 33971 | -0.517 | -0.0981 | Yes |
| 68 | FGFR1 | na | 34693 | -0.924 | 0.0010 | Yes |
Table: GSEA details [plain text format]

  

Fig 2: KEGG\_ADHERENS\_JUNCTION      
 Blue-Pink O' Gram in the Space of the Analyzed GeneSet

  

Fig 3: KEGG\_ADHERENS\_JUNCTION: Random ES distribution      
 Gene set null distribution of ES for **KEGG\_ADHERENS\_JUNCTION**
